# Effects of regular whey protein consumption on rat thyroid functions

**DOI:** 10.3906/sag-2010-270

**Published:** 2021-08-30

**Authors:** Gökhan AKKURT, Bahar KARTAL, Mustafa ALİMOĞULLARI, Sevil ÇAYLI, Ebru ALİMOĞULLARI

**Affiliations:** 1 Department of Surgical Oncology, Ankara City Hospital, Ankara Turkey; 2 Department of Histology Embryology, Medical College, Ankara Yıldırım Beyazıt University, Ankara Turkey; 3 Department of General Surgery, Yenimahalle Education and Research Hospital, Ankara Turkey

**Keywords:** Isole hydrolyzed whey protein, thyroid function, hyperthyroidism, hypothyroidism

## Abstract

**Background/aim:**

We aimed to investigate whether there was a significant difference in TSH, T3, T4 values and histopathologically evaluated thyroid tissues between rats that received ısole hydrolyzed whey protein (IHWP) at different doses regularly and rats fed with only standard feed.

**Material & methods:**

Total 24 rats were randomly divided into three groups with 8 rats in each group. First group were fed with standard feed for 12 weeks. Second group were given standard feed + daily 0.3 g/kg IHWP and rats in the third group standard feed + 0.5 g/kg IHWP for 12 weeks. Blood samples were collected from all rats before and after IHWP administration. All rats were then sacrificed, and thyroid tissues were histopathologically examined.

**Results:**

Interfollicular connective tissue areas and TSH (0.35–4.90 µIU/L) were higher in the control group compared to 3 cc IHWP and 5 cc IHWP groups, while thyroid hormone T4 (0.7–1.48 ng/dL), and thyroid hormone synthesis parameters including intrafollicular colloid amount, follicular diameter, and epithelial height were significantly higher in 3 cc and 5 cc IHWP groups compared to the control.

**Conclusion:**

We think that regular daily use of IHWP may increase the synthesis of thyroid hormone due to its high amino acid content.

## 1. Introduction

Thyroid hormones are involved in numerous vital functions in the body including total energy usage, cellular respiration, tissue growth, balance of nutrient and ion metabolism, and thermogenesis [1]. In order to maintain all these functions, thyroid hormones must be within a certain range in the blood [2]. The level of thyroid hormone (T3 and T4 levels) in the blood is controlled by the hypothalamus-pituitary-thyroid axis. The release of thyroid stimulating hormone (TSH), increases in cases of stress, disease, increased metabolic demand, and low levels of T3 and T4 [3]. Hormones such as somatostatin, dopamin, growth factor, and glucocorticoids can cause hypothyroidism [4]. The thyroid gland mainly consists of follicle clusters and follicles consist of single-layer epithelial cells with colloid in the lumen [5]. Tyrosine amino acid and iodine molecules are the main components of thyroid hormone. Synthesized thyroid hormones bind to thyroglobulin, stored in colloid and released into the bloodstream when necessary. Since thyroid hormones play a critical role in the maintenance of metabolism, various systems in the body are affected in the case of insufficiency or excess of these hormones [6]. The liquid part of milk, milk serum or whey is defined as whey protein (WP) [7]. Today, whey protein is commonly used due to its antioxidant, antitumoral, and immunity increasing features and its effect on protein synthesis [8]. There are few studies in the literature about the relationship between the use of whey protein supplements and thyroid functions. In a study by Carvalho et al., it was shown that uptake of exogenous amino acids, especially tryptophan, inhibit thyroid peroxidase activity. It was thought that this effect may occur due to the competition of exogenous amino acids with normal substrates for binding sites or reducing their oxidized forms[9]. In this study, we aimed to investigate whether IHWP has an effect on rat thyroid functions due to its intense amino acid contents in rats given daily IHWP supplement by gavage.

## 2. Material & methods

**Figure 1:**
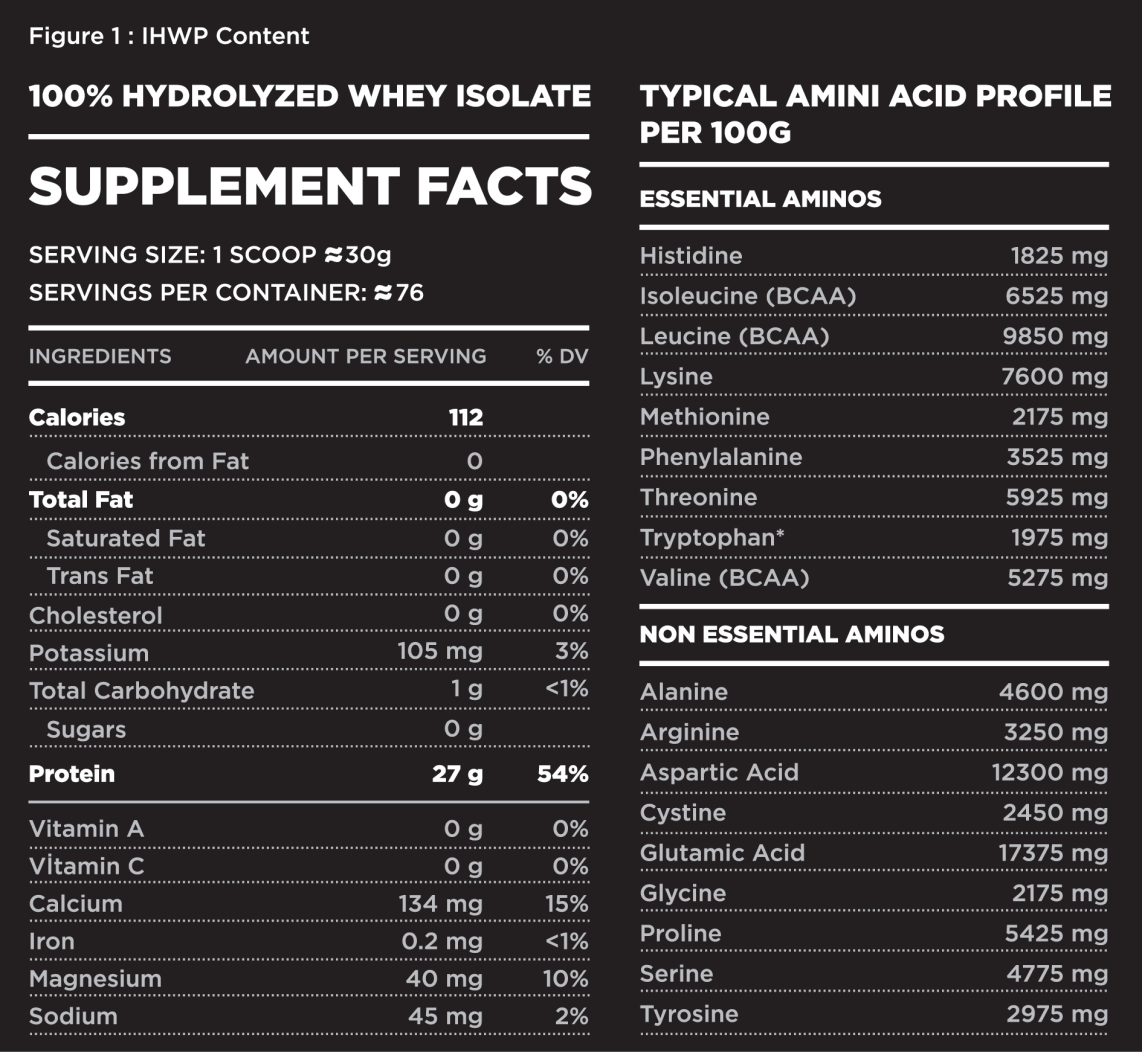
IHWP content.

This study was conducted in the Kobay Animal Breeding and Experimental Research Center. A total of 24, 4–6 weeks old Wistar Albino male rats, weighing 150–200 g were used in study for experimental purposes. Animals were observed for 10 days in order to provide adaptation to the experimental environment. The rats were divided into three groups with 8 rats in each group and housed in separate cages under a 12 h light/dark cycle. Rats in the first group were fed with standard feed for 12 weeks under laboratory conditions. Rats in group 2 and 3 daily received 0.3 g/kg and 0.5 g/kg IHWP (NAR Labs 100% hydrolyzed whey protein isolate 5 lb, USA) (Figure 1) via gavage respectively, in addition to standard feed for 12 weeks. Blood samples were taken from all rats before and 12 weeks after using IHWP to evaluate TSH, T3, T4 levels. At the end of the 12th week, anesthetic drugs xylazine 10 mg/kg and ketamine 200 mg/kg were administered to all rats and a kocher incision was performed. All rats were sacrificed and thyroid gland tissues were taken for histological evaluations. The intrafollicular colloid amount, follicular diameter, epithelial height and interfollicular connective tissue areas, degeneration in follicles, fibrosis, atypical follicle epithelium, mononuclear cell infiltration of thyroid gland tissues from each rat were histopathologically evaluated.

### 2.1. Whey protein

Whey is a complete protein containing 8 essential amino acids. It mainly contains betalactoglobulin (~ 65%), alpha-lactalbumin (~ 25%), albumin (~ 8%), and branched-chain amino acids such as leucine, isoleucine, and valine. It is also rich in glutathione precursors and tryptophan. Glutathione is a peptide containing 3 amino acids which are called gamma glutamic acid, cysteine, and glycine. Whey protein has three forms: hydrolyzed form, which is broken down to its constituents called hydrolyzate, isolated form in which all lactose has been removed, and concentrated form containing small amounts of fat and carbohydrates, produced by using ultrafiltration and diafiltration methods [10].

### 2.2. Histological examination

Thyroid tissues were fixed in 10% formaldehyde solution to examine under light microscopy. Tissue samples were washed with water to remove fixation solution from the samples. The samples were then dehydrated treated with graded alcohol series (70%–100%), made pellucid with xylene and blocked in paraffin. Sections of 5–6 µm were taken from the paraffin microtome. These sections were stained with hematoxylin and eosin (H&E) in order to examine tissues, thyroid follicular diameter, intrafollicular colloid amounts and the effect of colloid amounts on epithelial height. Six areas of 0.130 mm^2^ were randomly selected from each thyroid section stained with H&E using an imaging analysis system (Leica Q Win Standard). The diameter of the follicles that fit into this area and the area covered by the colloid in these follicles were measured and the amount of colloid was determined. Epithelial height was measured in 50 follicles randomly selected in each section.

### 2.3. Statistical analysis

The data were statistically analyzed with IBM SPSS Statistics v.23.0 software. Numerical variables were expressed as descriptive statistics (mean, standard deviation, median, quartiles). Differences between more than two groups were examined using Kruskal–Wallis test. As a result of the Kruskal–Wallis test, the Mann–Whitney U test was used to determine the groups that made a difference and Bonferronni correction was considered. As a result of the power analysis performed with the G power 3.1.9.2 program, the sample size was determined as 8 in each group of a total of 24 samples under the conditions of the first type error value was 0.05, the effect size was 0.7, and the power level was 0.80. p < 0.05 values were considered statistically significant.

## 3. Results

The study was conducted on 24 rats. There were differences between the groups in terms of interfollicular connective tissue area, TSH (0.35–4.90 µIU/L), T3 (1.71–3.71 pg/mL), T4 (0.7–1.48 ng/dL), intrafollicular colloid amount, follicular diameter, and epithelial height values (p < 0.05).

**Figure 2:**
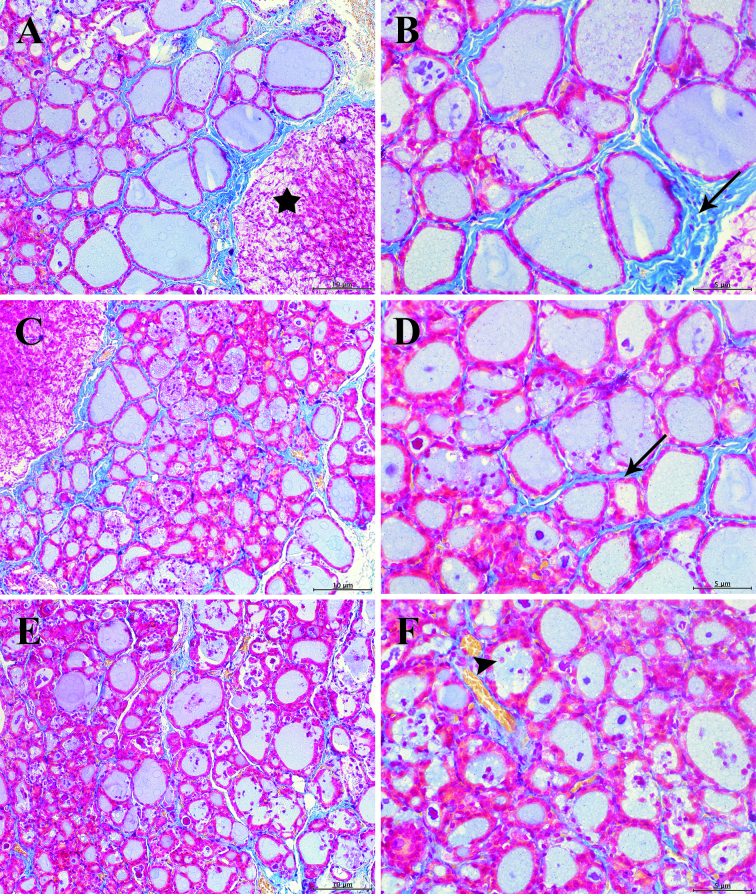
The representative photographs of rat tyroid tissue. Tyroid glands from rats: A,B: Control; C,D: 3 cc IHWP group; E,F: 5 cc IHWP; star: paratyroid gland; arrow: interfollicular collagen; arrow head: capillary (stained with Mallory Azan; left panel 20X and right panel for images at 40X magnification).

Accordingly, the mean interfollicular connective tissue area was higher in the control group (Figure 2a, Figure 2b, Figure 2c, Figure 2d) compared to 5 cc IHWP group (Figures 2e and 2f). The mean TSH (0.35–4.90 µIU/L) was higher in the control group compared to 3 cc IHWP and 5 cc IHWP groups, and the mean TSH (0.35–4.90 µIU/L) was higher in 3 cc IHWP group compared to 5 cc IHWP group.

The mean T3 (1.71–3.71 pg/mL) was lower in the control group compared to 5 cc IHWP groups, and the mean T3 (1.71–3.71 pg/mL) was lower in 3 cc IHWP groups compared to 5 cc IHWP group. The mean T4 (0.7–1.48 ng/dL) was lower in the control groups compared to both IHWP groups.

**Figure 3:**
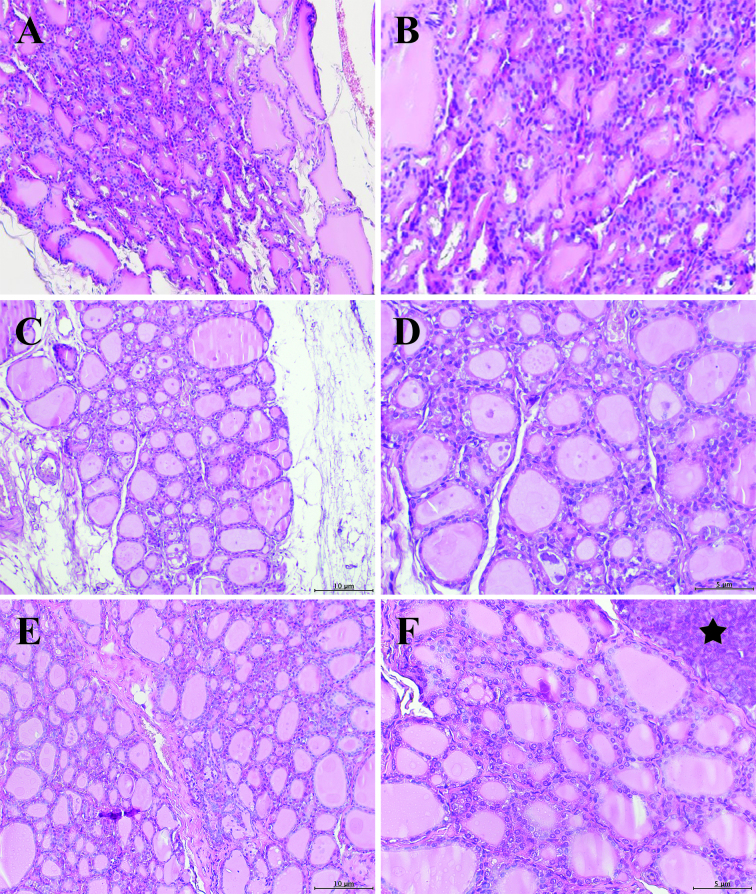
Histology of tyroid gland tissue. Tyroid glands from rats: A, B: Control; C, D: 3 cc IHWP group; E, F: 5 cc IHWP; star: paratyroid gland (stained with hematoxylin-eosin; left panel 20X and right panel for images at 40X magnification).

The mean intrafollicular colloid amount, follicular diameter, and epithelial height were lower in the control group (Figures 3a and 3b) compared to 3 cc IHWP (Figures 3c and 3d) and 5 cc IHWP groups (Figures 3e and 3f) , while the mean intrafollicular colloid amount, follicular diameter and epithelial height were lower in 3 cc IHWP compared to 5 cc IHWP groups (Tables 1 and 2). There were no differences between the groups in degeneration in follicles, fibrosis, atypical follicle epithelium, mononuclear cell infiltration of thyroid gland tissues (Table 3).

**Table 1:** Thyroid hormone difference between the groups.

		Mean ± sd	Median (25–75)	Test	p	Difference
TSH (0.35– 4.90)	Control	4.08 ± 0.43	4.00 (3.75–4.30)	17.175	0.000***	1>2>3
	3 cc	3.16 ± 0.45	3.05 (2.80–3.60)			
	5 cc	2.28 ± 0.64	2.45 (1.75–2.65)			
	Total	3.17 ± 0.90	3.15 (2.60–3.80)			
T3 (1.71–3.71)	Control	0.98 ± 0.18	0.95 (0.85–1.15)	16.509	0.000***	1,2<3
	3 cc	1.53 ± 0.46	1.60 (1.15–1.90)			
	5 cc	2.40 ± 0.37	2.45 (2.05–2.75)			
	Total	1.63 ±0.69	1.60 (1.05–2.15)			
T4 (0.7–1.48)	Control	0.71 ± 0.21	0.65 (0.55–0.85)	10.637	0.005**	1<3
	3 cc	0.93 ± 0.18	0.90 (0.80–1.05)			
	5 cc	1.14 ± 0.21	1.15 (1.00–1.30)			
	Total	0.93 ± 0.26	0.90 (0.75–1.10)			

*: p < 0.05, **: p < 0.01, ***: p < 0.001.

**Table 2:** Differences of thyroid tissue histopathological findings between groups.

		Ort ± ss	Median (25–75)	Test	p	Difference
Interfollicular connective tissue area	Control	2681.87 ± 1697.61	2540.52 (1673.93–3446.80)	9.555	0.008**	1>3
	3 cc	1616.45 ± 1458.23	1091.29 (752.88–1840.93)			
	5 cc	652.61 ± 580.83	483.49 (248.48–947.65)			
	Total	1650.31 ± 1530.91	1280.39 (570.99–2435.06)			
Intrafollicular colloid amount	Control	76.52 ± 23.65	76.89 (58.28– 96.49)	16.980	0.000***	1<2,3
	3 cc	147.11 ± 12.33	150.50 (138.20–152.86)			
	5 cc	165.29 ± 19.17	157.33 (150.75–181.49)			
	Total	129.64 ± 43.11	148.63 (96.49–154.96)			
Follicular diameter	Control	92.10 ± 19.87	100.39 (77.28–105.48)	15.540	0.000***	1<2,3
	3 cc	183.63 ±14.26	184.35 (174.51–189.16)			
	5 cc	197.71 ± 25.28	188.94 (179.07–215.82)			
	Total	157.81 ± 51.62	179.07 (105.48–189.16)			
Epithelialheight	Control	5.38 ± 1.00	5.66 (4.92–5.89)	18.604	0.000***	1<2<3
	3 cc	9.13 ± 2.29	8.22 (8.00–10.99)			
	5 cc	14.74 ± 1.90	15.06 (14.20-15.79)			
	Total	9.78 ± 4.36	8.22 (5.79-14.23)			

*: p < 0.05, **: p < 0.01, ***: p < 0.001

**Table 3:** Differences of thyroid tissue histopathological scoring between groups.

	Control	3 cc	5 cc
	0 1 2 3	0 1 2 3	0 1 2 3
Degeneration in follicles	8	8	8
Fibrosis	8	8	8
Atypical follicle epithelium	8	8	8
Mononuclear cell infiltration	8	7 1	7 1

(0) score, no structural damage; (1) score, minimal damage; (2) score, moderate damage; (3) score, serious damage.

## 4. Discussion

Thyroid hormones are necessary for normal development of vertebrate. Hypothalamic–pituitary–thyroid axis (HPT) protect the euthyroid state. One of the effective environmental factors determining the activity of HPT axis is nutrition. Serum thyroid hormone levels are strictly regulated with a negative feedback mechanism in HPT axis, which involves hypothalamus, pituitary and thyroid glands [11]. The regulation of HPT axis with thyrotropin-releasing hormone (TRH) neurons is important for the adaptive changes in the activity of thyroid axis in response to external and internal stimuli [12]. TRH and TSH-β gene expression and the level of thyroid hormone in circulation decreases during fasting in humans [13]. Decreased protein content in diets changes the activity of HPT axis at both central and peripheral levels similar to the effect caused by fasting. In a study by Shi et al. a significant decrease was observed in hypothalamic TRH, pituitary TSH-β transcript, and plasma T3 concentration in the rats fed with a diet not containing protein compared to the rats fed with a control diet [14]. In our study, we found that a high protein-containing nutrition increased T3 hormone. In another study, it was demonstrated that the gene expression of hypothalamic NPY in the rats fed with a protein-restricted diet was close to the level of energy-restricted animals and was significantly higher than in rats fed with high-protein, low-carbohydrate and low-fat diet [15]. In a study by Shrader et al. it was found that the thyroid structure of fetuses and newborns was affected as a result of protein malnutrition, causing a decrease in thyroid volume due to the delay in follicular organization. The authors concluded that these results caused decreases in the number of follicles and thyroid size [16]. In addition, in another study by Gao et al. association of low protein level and excess iodine level produced a diffuse cellular damage in the thyroid gland only due to excess iodine dose [17]. Likewise, in our study, it has been observed in recent studies that low protein and high-carbohydrate diets increase serum T3 concentrations and decrease free T3, T4, and TSH concentrations. In accordence with Lunn and Austin, T3 does not increase in low protein and carbohydrate intake, while it increases in low protein and high carbohydrate intake. This was attributed to the increased affinity of T3 binding by thyroid hormone transport proteins due to unbalanced consumption of macronutrients [18,19]. Source of the consumed protein has also an important role on thyroid hormone concentration. There are studies reporting that excitatory amino acid (EAA) such as glutamate and aspartate can change hormone secretion from the pituitary–thyroid axis [20]. In a study by Alfonso et al. intraperitoneally administered glutamate (l-Glu, 20 and 25 mg/kg) and n-methyl-d-aspartate (NMDA, 25 mg/kg) increased serum T3, T4, and TSH concentrations in adult male rats [21]. In another study, it was shown that isoflavones that are the major component of soybean affects the activity of thyroid axis both in vitro and in vivo, and Genistein, which is one of the soy isoflavones is a potent inhibitor of thyroid peroxidase (TPO) activity [22]. TPO is a membrane-bound enzyme, which contains glycoprotein and plays an important role in the biosynthesis of thyroid hormones. Iodure oxidation, thyroglobulin iodination, and binding of iodotyrosine remnants of thyroglobulin are catalyzed by TPO [23]. In a study by Carvalho et al. normal in vivo TPO iodure oxidation activity was shown to be completely catalyzed by a hydrolyzed TPO preparate (0.15 mg/mL) and hydrolyzed bovine serum albumin (BSA, 0.2 mg/mL). Some amino acids such as pancreatic casein hydroxylate, cysteine, methionine, and tryptophan completely inhibited TPO mediated iodure oxidation reaction, while tyrosine, phenylalanine and tryptophan inhibited this reaction by 54%. It was thought that inhibitor agents interfered with enzyme activity by competing with their normal substrates for binding sites, decreasing free substrates, or reducing oxidized forms[9]. Tyrosine is one of 22 amino acids in the body, and functions in protein synthesis. The most important function of tyrosine is being a precursor amino acid in the synthesis of dopamine, norepinephrine, epinephrine, melanine, and thyroxine. Tyrosine is found in high-protein foods and its synthesis occurs with degradation in the liver [24]. Tyrosine is involved in the synthesis of some hormones in the body, while some diseases may occur with its deficiency. Iodine entering into the thyroid gland combines with tyrosine here and forms thyroid hormones (T3, T4) [25]. In addition, numerous studies have been conducted on the relationships of tyrosine with stress, fatigue, prolonged sleep disorder, stress induced weight loss, stress induced changes in blood pressure, neuroendocrine system, perceptive, cognitive and physical performance in humans, and stress hormones [26]. In a study by Sarıkaya et al. methyl-tyrosine enhancement in the pancreas was found to be higher than in the liver and it was shown that L-tyrosine compound ^131^I was marked at a high rate, its enhancement in the stomach, kidneys, pancreas, and thyroid was high and it has a stability enough for diagnostic investigations [27].

Whey is a by product which formed during the production of cheese and casein. Powder forms of whey are commonly used in the food industry and high-protein foods produced for infants. In addition, it is also used by athletes especially dealing with body building worldwide in order to increase muscle mass [28]. Concentrated whey protein usually contains 80% protein (78.2%), 0.5% fiber, 8% carbohydrate, about 7% fat as well as amino acids, growth factors, and cytokines. Ingredients vary among whey protein types in the market, and today especially IWHP is preferred due to its rapid absorption feature [29]. In recent years, many studies have been conducted to reveal the role of nutrients in prevention and treatment of diseases [30]. Studies have reported that whey proteins have antidiabetic, blood pressure lowering, cardiovascular system function improving, antibacterial, and antiviral activities [31]. According to our observations, although there are numerous studies about the benefits of IHWP usage, the number of studies investigating side effects of regular IHWP usage is limited. In this study, for the first time we observed that daily given oral IHWP supplement increased thyroid hormones T3 and T4, and intrafollicular colloid amounts, follicular diameter, and epithelial height that are the markers of thyroid hormone synthesis in the thyroid tissue.

The use of IHWP, especially to enhance muscular development may have benefits for the body as well as may cause various changes in thyroid functions, particularly hepatic and renal functions due to prolonged usage. In our study, especially high oral tyrosine and tryptophane intake was found to increase thyroid hormones. We think that regular daily use of IHWP may increase the synthesis of thyroid hormone and persons using IHWP supplement should have thyroid function tests in certain periods. 

## Informed consent

This study was conducted in the Kobay Animal Breeding and Experimental Research Center and approved by the local ethics committee (Approval number: 375).
